# Editorial: Radiomics and artificial intelligence in radiology and nuclear medicine

**DOI:** 10.3389/fmed.2023.1216434

**Published:** 2023-05-23

**Authors:** Salvatore Annunziata, Giorgio Treglia

**Affiliations:** ^1^Unità di Medicina Nucleare, GSTeP Radiopharmacy - TracerGLab, Dipartimento di Diagnostica per Immagini, Radioterapia Oncologica ed Ematologia, Fondazione Policlinico Universitario A. Gemelli, IRCCS, Rome, Italy; ^2^Division of Nuclear Medicine, Imaging Institute of Southern Switzerland, Ente Ospedaliero Cantonale, Bellinzona, Switzerland; ^3^Faculty of Biomedical Sciences, Università della Svizzera italiana, Lugano, Switzerland; ^4^Faculty of Biology and Medicine, University of Lausanne, Lausanne, Switzerland

**Keywords:** radiology, artificial intelligence, radiomics, machine learning, nuclear medicine, imaging, deep learning

Artificial intelligence (AI) and radiomics algorithms in radiology and nuclear medicine have demonstrated a good performance as diagnostic, predictive or prognostic markers for several diseases with a high potential to be used as clinical tools. However, these algorithms should be further validated in clinical practice to spread their routinary use worldwide.

After the successful publication of a previous Research Topic about Radiomics in Positron Emission Tomography (PET) on Frontiers in Medicine in 2021 (https://www.frontiersin.org/research-topics/15427/artificial-intelligence-in-positron-emission-tomography) a wider and more comprehensive Research Topic on the role of AI and radiomics in radiology and nuclear medicine was launched in 2022. This Research Topic comprises 12 articles.

For successful employment of AI and deep learning algorithms in the clinical practice, explainable artificial intelligence (XAI) could be introduced for several imaging modalities as clearly discussed in the comprehensive review of De Vries et al. including 75 articles (De Vries et al.). However, the authors demonstrated that there is currently no clear consensus on how XAI should be used in order to close the gap between medical professionals and deep learning algorithms for clinical implementation. Furthermore, De Vries et al. also suggested a systematic technical and clinical quality assessment of XAI methods.

Seven articles included in this Research topic are dedicated to the use of AI in oncological imaging.

Radiomic features could be very useful for a better prognostic stratification in patients with glioblastoma. The study of Chiesa et al. including 90 patients with glioblastoma applied a radiomic analysis focusing on healthy tissue ring around the surgical cavity on post-operative magnetic resonance imaging. This study provided a preliminary model for a decision support tool for a customization of the radiation target volume in glioblastoma patients to achieve a margin reduction strategy (Chiesa et al.).

Sun et al. assessed the value of radiomics based on computed tomography (CT) images in the preoperative discrimination between lung invasive adenocarcinomas and non-invasive adenocarcinomas among 1,185 pulmonary nodules. The authors found that radiomics based on CT images showed good predictive performance in discriminating between these tumoral entities, especially in part solid nodule group. Furthermore, radiomics based on contrast enhanced CT images provided no additional value compared to non-contrast enhanced CT images (Sun et al.).

A brief research report evaluated the performance of fluorine-18 fluorodeoxyglucose ([^18^F]FDG) PET/CT radiomic features to predict overall survival in 50 patients with locally advanced uterine cervical carcinoma. The authors found that standardized uptake value peak (SUVpeak) and the textural feature gray-level run-length matrix (GLRLM) presented the best performance to predict overall survival in patients with cervical cancer undergoing chemo-radiotherapy and brachytherapy (Goncalves de Alencar at al.).

A retrospective study assessed the predictive ability of [^18^F]FDG PET/CT radiomic features for MYCN, 1p and 11q abnormalities in 122 pediatric patients with neuroblastoma. The authors clearly demonstrated that baseline [^18^F]FDG PET/CT radiomics is able to predict MYCN amplification and 1p and 11 aberrations in patients with neuroblastoma, thus aiding tumor staging, risk stratification and disease management (Qian et al.).

Another retrospective study on 131 patients explored the application of [^18^F]FDG PET/CT radiomics in the identification and correct classification of spine multiple myeloma lesions and bone metastases. The radiomics model constructed based on [^18^F]FDG PET/CT images achieved satisfactory diagnostic performance for the classification of multiple myeloma and bone metastases. In addition, the radiomics model showed significant improvement in diagnostic performance compared to human experts and PET conventional parameters (Jin et al.).

A retrospective study from Morland et al. estimated the ability of a new index, uptake formula, including healthy organs standardized uptake values on [^18^F]FDG PET/CT to predict event free survival in 163 patients with Hodgkin lymphoma. The Uptake Formula showed a similar performance to total metabolic tumor volume in predicting event free survival in Hodgkin lymphoma (Morland et al.).

Ghezzo et al. tested on a cohort of 85 prostate cancer patients a recently proposed convolutional neural network for the automatic segmentation of intraprostatic cancer lesions on prostate specific membrane antigen PET images. The authors demonstrated that the AI model could be used to automatically segment intraprostatic cancer lesions to define the volume of interest for radiomics or deep learning analysis. However, more robust performance is needed for the generation of AI-based decision support technologies to be proposed in clinical practice (Ghezzo et al.).

Beyond oncological indications of imaging methods, AI may be also applied for other indications. For instance, the review article of Yan et al. have summarized the application of radiomics for predicting recurrent pancreatitis, evaluating the clinical severity of pancreatitis, differentiating pancreatitis from pancreatic adenocarcinoma, and functional abdominal pain from pancreatitis, identifying pancreatis, its risk factors and complications (Yan et al.).

Flaus et al. developed a deep learning-based [^18^F]FDG PET image enhancement method using simulated brain PET to improve visualization of epileptogenic lesions. However, the authors recommended further evaluation to generalize their method and to assess its clinical performance in a larger cohort (Flaus et al.).

Weakly supervised deep learning models have gained increasing popularity in medical image segmentation. However, these models are not suitable for the critical characteristics presented in chest radiographs: the global symmetry of chest radiographs and dependencies between lesions and their positions. In their study, Gu et al. proposed a weakly supervised model, Chest L-Transformer, to take these characteristics into account. The authors demonstrated a significant segmentation performance improvement over the current state-of-the-art while achieving competitive classification performance (Gu et al.).

Lastly, an original article by Quak et al. including 67 patients demonstrated that the degradation of image quality on PET due to a reduction in injected activity at the end of the ^68^Ge/^68^Ga generator lifespan can be effectively counterbalanced by using AI-based PET denoising (Quak et al.).

Finally, we would like to underline that AI and radiomics tools are widely used for research purpose in the fields of radiology and nuclear medicine. Nevertheless, large validation protocols and real-life experience are needed to allow an increasing use of these tools in clinical practice, with possible benefit for patients' treatments and outcomes.

## Author contributions

SA and GT drafted the manuscript and revised the final version. All authors contributed to the article and approved the submitted version.

